# Validation of Administrative Osteoarthritis Diagnosis Using a Clinical and Radiological Population-Based Cohort

**DOI:** 10.1155/2016/6475318

**Published:** 2016-12-29

**Authors:** M. Mushfiqur Rahman, Jacek A. Kopec, Charlie H. Goldsmith, Aslam H. Anis, Jolanda Cibere

**Affiliations:** ^1^Department of Applied Statistics, East West University, Aftabnagar, Dhaka 1212, Bangladesh; ^2^School of Population and Public Health, University of British Columbia, Vancouver, BC, Canada; ^3^Arthritis Research Centre of Canada, 5591 No. 3 Road, Richmond, BC, Canada V6X 2C7; ^4^Health Sciences, Simon Fraser University, Burnaby, BC, Canada; ^5^Centre for Health Evaluation and Outcome Sciences, Vancouver, BC, Canada; ^6^Department of Medicine, University of British Columbia, Vancouver, BC, Canada

## Abstract

*Objectives.* The validity of administrative osteoarthritis (OA) diagnosis in British Columbia, Canada, was examined against X-rays, magnetic resonance imaging (MRI), self-report, and the American College of Rheumatology criteria.* Methods.* During 2002–2005, 171 randomly selected subjects with knee pain aged 40–79 years underwent clinical assessment for OA in the knee, hip, and hands. Their administrative health records were linked during 1991–2004, in which OA was defined in two ways: (AOA1) at least one physician's diagnosis or hospital admission and (AOA2) at least two physician's diagnoses in two years or one hospital admission. Sensitivity, specificity, and predictive values were compared using four reference standards.* Results.* The mean age was 59 years and 51% were men. The proportion of OA varied from 56.3 to 89.7% among men and 77.4 to 96.4% among women according to reference standards. Sensitivity and specificity varied from 21 to 57% and 75 to 100%, respectively, and PPVs varied from 82 to 100%. For MRI assessment, the PPV of AOA2 was 100%. Higher sensitivity was observed in AOA1 than AOA2 and the reverse was true for specificity and PPV.* Conclusions.* The validity of administrative OA in British Columbia varied due to case definitions and reference standards. AOA2 is more suitable for identifying OA cases for research using this Canadian database.

## 1. Introduction

Osteoarthritis (OA) is one of the most prevalent chronic health conditions that causes disability among the elderly [1, 2]. While the prevalence of OA in the general population depends on the joint sites, diagnostic methods, sex, age range, and geographic region, approximately 10–12% of the global population have OA [3–6]. In epidemiologic research, there is no simple way to define the presence or absence of OA or to distinguish between incident and progressive disease. However, an accurate estimate is necessary for the policy makers and healthcare professionals to improve the health condition of OA patients through disease management and public health programs [4, 5, 7–9]. In the British Columbia (BC) administrative database, the overall prevalence rate of OA in any joint was 10.8% in 2001 [3]. Other international studies reported the prevalence of radiographic, symptomatic, and self-reported OA in the knee, hip, and hand joints [2, 6, 10–12].

The most common way to diagnose OA cases is the radiographic examination using Kellgren-Lawrence (K-L) grading system [13]. Other methods include magnetic resonance imaging (MRI) [14, 15] and self-reporting [10]. Knee, hand, and hip OA are also assessed using the American College of Rheumatology (ACR) clinical criteria [16–18]. Administrative health records represent useful resources for chronic disease surveillance because the data are routinely collected, cover wide geographic areas, and capture the great majority of the subjects registered in the healthcare system. Recently, these databases have been frequently used for health research, where OA cases are identified on the basis of several definitions using International Classification of Disease (ICD) codes [3, 19, 20]. Utilizing these data requires assessing the validity of case definitions. The accuracy of administrative OA case definitions has been validated in previous studies against self-reported population surveys [21] and medical records [19]. However, these studies covered only 2–5 years of observation and did not include MRI assessments.

In this study we aimed to examine the validity of OA diagnoses recorded in the BC administrative database. Our primary objective was to determine the sensitivity, specificity, positive predictive value (PPV), negative predictive value (NPV), and likelihood ratios of two administrative case definitions of OA. We examined the accuracy of these definitions using four reference standards that include X-rays, MRI, self-reports, and the ACR clinical criteria. Evaluating the validity of administrative OA diagnoses is an important step in conducting further research using these databases.

## 2. Materials and Methods

### 2.1. Data Source

A cohort of 255 subjects with knee pain was recruited through population random sampling from Vancouver, BC, during the period August 2002 to February 2005. The subjects met inclusion criteria if they were between 40 and 79 years of age and had pain, aching, or discomfort in or around the knee at any time in the past 12 months. Subjects who had inflammatory arthritis, fibromyalgia, knee arthroplasty, a history of knee surgery/injury within the past 6 months, knee pain referred from the hip or back, and inability to undergo MRI were excluded. From the greater Vancouver telephone directory, 5,231 English-speaking persons were randomly contacted, of whom 3,269 (62.5%) agreed to participate in the survey. From the 3,269 subjects, 91.9% were ineligible due to age restriction and other exclusion criteria. Of the remaining 265 subjects, 10 were excluded due to missed appointments and for other reasons. The study sample recruitment procedure has been described elsewhere [22]. Finally, 255 selected subjects underwent comprehensive clinical assessment, standardized joint examination, X-rays, and MRI to identify knee OA. Of the 255 subjects, clinical data on 171 were linked with the administrative health records for the period 1991–2004 through personal health numbers, because written consents were available for only these subjects. The BC Ministry of Health approved access to and use of the data facilitated by Population Data BC for this study. This administrative database consists of linkage of the Medical Service Plan (MSP) payment information for the period 1990/91–2003/04 [23], the PharmaCare data for the period 1990–2004 [24], and Hospital separation records for the period 1990/91–2003/04 [25]. Administrative database includes information on date of birth, sex, physician billing information for any health consultation, socioeconomic status by area of residence, hospital diagnoses, dates of hospital admissions, the 9th and 10th revisions of the ICD codes (ICD-9 and ICD-10, resp.), and death records of all individuals registered in the Medical Service Plan (MSP) of BC. MSP is a publicly funded plan in which approximately 99% of BC residents are registered. The study was approved by the Clinical Research Ethics Board at the University of British Columbia, Canada.

### 2.2. Administrative Definition of OA

Administrative OA was defined in two ways based on ICD-9 and ICD-10 codes, referred to as AOA1 and AOA2. AOA1 required at least one visit to a health professional or one hospital admission with the ICD-9 code of 715 or the ICD-10 codes from M15 to M19, and AOA2 required at least two visits to health professionals in two years separated by at least one day or one hospital admission with these codes. For AOA2, the date of the second qualifying visit was used to assign the diagnosis date. These ICD codes include symptomatic and radiographic OA in any joint except the spine. The most commonly used pain medications for OA treatment are acetaminophen and nonsteroidal anti-inflammatory drugs [8, 26]. Often these medications are available over the counter and require no prescriptions. Thus, it is not appropriate to include the history of pain medication use in OA case definitions.

### 2.3. Knee, Hand, and Hip OA Assessment

Knee OA was assessed with a comprehensive questionnaire which included duration of knee pain, frequency of pain (number of days over the past month), and pain location using a knee diagram [27]. A standardized knee examination was performed by a rheumatologist [28]. The ACR clinical criteria for knee OA [16] include pain in the knee and any three of the following: (1) over 50 years of age, (2) less than 30 minutes of morning stiffness, (3) crepitus on active motion, (4) bony tenderness, (5) bony enlargement, and (6) no palpable warmth. The presence of hand OA was determined by using ACR criteria for hand OA, which included pain, aching, or stiffness in the hand and any three of the following conditions: (1) hard tissue enlargement of two or more of the following joints: 2nd and 3rd distal interphalangeal, the 2nd and 3rd proximal interphalangeal, and the 1st carpometacarpal joints of both hands; (2) hard tissue enlargement of 2 or more distal interphalangeal joints; (3) less than three swollen metacarpophalangeal joints; (4) deformity of 2 or more joints listed in (1) [17]. Although ACR criteria for hip OA include pain in the hip and any two of the following: (1) ESR < 20 mm/hour, (2) radiographic femoral or acetabular osteophytes, and (3) radiographic joint space narrowing [18], only hip pain was assessed in our study.

### 2.4. Radiographic K-L Grade

Knee radiography was completed within a month of the clinical assessment. Details on X-ray procedures have been described previously [22, 29]. X-rays were scored using the K-L 0–4 grading system [13] independently by 2 readers who were blinded to the clinical and MRI information. The intraclass correlation coefficient was 0.79 and the differences in readings were adjudicated by consensus readings by the 2 readers. Subjects were classified as having radiographic OA if their K-L grade was greater than or equal to 2.

### 2.5. MRI Cartilage Score

MRI for the most painful knee was performed within a month of clinical assessment. Detailed information regarding how MRI was performed has been described previously [22]. Briefly, six joint areas were assessed, including the medial and lateral tibial plateau and femoral condyles, patella, and trochlear groove. Cartilage was graded on a semiquantitative scale of 0–4 based on the following definitions: 0 = normal, 1 = abnormal signal without a cartilage contour defect, 2 = contour defect of less than 50% cartilage thickness, 3 = contour defect of 50–99% cartilage thickness, and 4 = 100% cartilage contour defect with subjacent bone signal abnormality [30, 31]. The MRIs were read by a single reader, who was blinded to the radiographic and clinical information. The intrarater reliability of the cartilage readings was high, varying from 0.84 to 1.0 for different cartilage surfaces. Based on the MRI cartilage scores, subjects were classified as having knee OA if the score was greater than or equal to 2.

### 2.6. OA by Self-Report

In the baseline questionnaire, knee OA was assessed by asking two questions. (1) “Has a doctor ever told you that you have osteoarthritis (also called degenerative or wear-and-tear arthritis) in your right knee?”, and (2) “has a doctor ever told you that you have osteoarthritis (also called degenerative or wear-and-tear arthritis) in your left knee?” Pain in the hip joints was assessed by the following instruction: “In the following homunculus diagram each circle represents a joint. Please mark each joint where you have experienced pain or discomfort over the past 12 months.” We counted subjects if they marked in the hip joints in the homunculus diagram.

### 2.7. Reference Standard

For the selected subjects, knee OA was assessed based on the above four measurements. In addition, hand and hip OA were assessed using the ACR clinical criteria and the self-reported hip pain, respectively. Based on the knee, hand, and hip OA assessments, we defined four reference standards: RS1, RS2, RS3, and RS4. RS1 included assessments of knee and hand OA based on the ACR clinical criteria and hip OA based on self-reported hip pain. RS2 included assessments of knee, hand, and hip OA based on K-L grade, ACR clinical criteria, and self-reported hip pain, respectively. RS3 included assessments of knee, hand, and hip OA based on MRI cartilage score, ACR clinical criteria, and self-reported hip pain, respectively. RS4 included assessments of knee, hand, and hip OA based on self-reports, ACR clinical criteria, and self-reported hip pain, respectively. The same measurements for hand and hip OA were consistently included in the four reference standards.

### 2.8. Statistical Analysis

Baseline characteristics of the cohort were age, body mass index (BMI) (kg/meter^2^), hip pain, symptomatic hand OA, and pain medication used. These characteristics were determined separately for men and women. We calculated the sensitivity, specificity, PPV, and NPV, for each case definition according to four reference standards. The 95% confidence intervals (CIs) were calculated for these statistics. For more detail about these measures, please refer to Rothman et al. [32]. In addition, we have calculated likelihood ratios (LR+ and LR−) and their 95% CIs, with LR+ = positive likelihood ratio = sensitivity/(1 − specificity), and LR− = negative likelihood ratio = (1 − sensitivity)/specificity. All analyses were performed using SAS V.9.3 (SAS Institute, Cary, NC, USA).

## 3. Results

Characteristics of 171 subjects by sex are presented in Table 1. The mean age of the subjects was 59 years, and 51% were men. The BMI ranged from 19 to 43 and men were more overweight and obese than women (*p* value = 0.02). Hip pain and hand OA were more common in women than in men (*p* value < 0.01). Statistically significant differences between men and women were observed for the proportion diagnosed with OA by each of the four reference standards except for RS3. Among the four different knee OA measurements, MRI detected the highest percentages of OA (91.7% in women and 88.5% in men) and X-rays detected the lowest percentages of OA (42.9% in women and 44.9% in men).

The validation results of two administrative OA definitions compared to the four reference standards are presented in Table 2. The sensitivity of case definitions AOA1 and AOA2 varied from 47 to 57% and 21 to 26%, respectively. Higher sensitivity was observed in AOA1 compared to AOA2 and the highest sensitivity (95% CI) was 57% (48–66%) for AOA1 when the reference standard included self-reported physician diagnosed knee OA. The specificity varied from 75 to 87% for AOA1 and from 91 to 100% for AOA2. The highest specificity (95% CI) was 100% (70–100%) for AOA2 when the reference standard included MRI of the knee OA. PPVs varied from 82 to 96% for AOA1 and from 85 to 100% for AOA2. The lowest NPV (95% CI) was 9% (5–15%) for AOA2 when the reference standard included MRI score for knee OA. The positive likelihood ratio (LR+) was greater than 5 in AOA2 for the reference standards RS3 and RS4, and therefore AOA2 may be useful in ruling in OA. On the other hand, values of negative likelihood ratio (LR−) were between 0.5 and 0.8. Therefore, these definitions may not be very useful to ruling out OA [33].

## 4. Discussion

Based on the BC administrative health records, we have assessed the validity of two case definitions of OA using four reference standards. The reference standards included radiographic K-L grade, MRI cartilage scores, self-reports, and the ACR clinical criteria for the knee OA assessments, the ACR clinical criteria for the hand OA assessments, and self-reported hip pain records for the hip OA assessments. Of the two administrative definitions, AOA1 had the higher sensitivity and NPV whereas AOA2 had the higher specificity and PPV. Validity measures were similar among the four reference standards in each case definition, while both case definitions of OA yielded a PPV of more than 82%.

Our validation results are comparable with those obtained in Lix et al.'s [21] study in which self-reported survey data were used as a reference standard. Using two years of data and the definition of at least two physician's diagnoses or one hospital separation, the authors obtained a sensitivity of 42.6% and a specificity of 88.1%. For the definition based on one physician's diagnosis, they obtained a higher sensitivity but a lower specificity, which is consistent with our results. The administrative health records may include some individuals whose OA has gone undiagnosed during the observation period. This could potentially contribute to the lower-than-expected sensitivities in both case definitions. After examining the medical history of OA cases over a period of two years, Harrold et al. [19] obtained a PPV of 62% for administrative OA diagnoses. The likely reason why we obtained higher PPVs was that we used 13 years of administrative records and the prevalence of OA was higher in our cohort. In our cohort, the majority of the subjects had preradiographic disease (K-L < 2); we observed that 90% of these symptomatic subjects had knee OA based on MRI cartilage assessment. In contrast to X-rays, MRI can detect preradiographic as well as radiographic OA in the knee and other joints [15, 34]; consequently, higher specificity and PPV were obtained when MRI knee assessment was used as the reference standard. In validation studies, PPV and NPV depend on the prevalence and severity of the disease. Thus, in addition, we have calculated positive and negative likelihood ratios, which are independent of the prevalence. On the basis of likelihood ratios, AOA2 might be useful in ruling in OA.

The limitations of the present study need to be acknowledged. First, we received written consent from 171 subjects to link their clinical data with the administrative records, which reduced the sample size. This reduction slightly changed the sample characteristics compared to those of the entire cohort [22]. Second, some of these subjects were in the early stage of OA development. The recruitment period for subjects was 2002–2005, and their administrative histories were linked from 1991 to 2004. In an ideal situation, both clinical and administrative diagnoses should have been performed in the same calendar year. However, among the elderly with OA and other chronic diseases, the former often receives lower priority when they are assessed by a physician. Therefore, the number of OA cases covering 2-3 years of administrative records is expected to be lower than the actual number of cases. To minimize the number of undiagnosed OA cases we observed the medical history of these subjects from 1991 to 2004. We did not include administrative records after the clinical assessment to reduce false positives. Third, we used hip pain as a proxy variable for hip OA in the reference standards. Studies have shown that hip pain is considered to be the main feature of hip OA [35, 36]. The knee, hip, and hand are the most commonly affected joints [2, 6, 10–12] and studies have shown that individuals with OA in one joint are more likely to have the disease in other joints [37]. By including hip OA cases based on hip pain, we added 1–11% additional OA cases to the reference standards, which may not overrepresent the actual hip OA cases. Fourth, OA in other locations, such as the foot, elbow, jaw, and shoulder, were not measured in the reference standards. This is unlikely to have a substantial effect on the validation results since the prevalence of OA in these locations is relatively low. Fifth, our study subjects were selected based on knee pain. Future validation studies of randomly selected subjects with symptomatic OA in any joint, as well as comparing clinical diagnoses of OA in all possible joints with administrative diagnoses, are needed. We have validated two commonly used case definitions of OA in this study. Validation studies focusing on other administrative definitions or algorithms for OA might be the subject of future studies.

The strengths of this study include the use of a representative clinical sample linked to administrative data. Our study featured a population-based cohort that included subjects with preradiographic as well as advanced radiographic knee OA. We compared two administrative OA definitions to the four reference standards. To our knowledge, this is the first study, to compare administrative case definitions and MRI-detected cartilage-based OA assessments. Administrative databases are frequently used in OA research. However, there are few validation studies of administrative OA diagnosis. The primary objective of selecting this study cohort was to assess MRI, X-rays, and symptomatic-based measures to detect early knee OA. In addition, symptomatic and self-reported data were collected for hand and hip OA, which enhances the present study. In a site-specific validation study focusing one joint at a time, the validation results may vary between sites. Since administrative diagnosis includes OA in any joint except the spine, our validation results are not affected by site-specific variations.

Population-based administrative data have great potential for facilitating investigations of OA occurrence as well as OA comorbidity and outcome research. However, the fundamental question to be addressed is whether the data are valid for such purposes. Our study addressed this question by comparing two case definitions with four reference standards. The next question to be addressed is which case definition should be applied for defining OA? It is noteworthy that the observed PPVs in both definitions were very high because the prevalence of OA was more than 70% based on the reference standards, whereas, in the general population, the prevalence of OA is 10–20%. The sensitivity of the definition that included one physician's claim or hospital admission was 47–57%, and the specificity was 75–87%. This suggests that potential overreporting should be a concern in estimating the general population prevalence using this definition. On the other hand, the sensitivity of the definition that included at least two physician's claims in two years or one hospital admission was 21–26%, and the specificity was 91–100%. This suggests that prevalence would likely be underreported using the latter definition. In addition, the observed specificity and the PPV in the latter case definition were higher than those in the former case definition, thus producing fewer false positives cases. The definition of at least two physician's claims in two years or one hospital admission would, therefore, be more appropriate for studies in which avoiding false positives is critical, such as etiological research or studies assessing the effect of OA on other health conditions in the population.

In conclusion, the validity of OA diagnoses in administrative health records in British Columbia varied due to case definitions and reference standards. AOA2 is more suitable for identifying OA cases for research using this Canadian administrative database. Despite several limitations, we have validated two administrative case definitions wherein clinical and symptomatic diagnoses of knee, hand, and hip OA were included in the reference standards. Future validation studies, based on clinical diagnoses of all possible joints affected by OA, are needed. As the validation results may differ across administrative regions, further studies in different populations are needed to compare these results.

## Figures and Tables

**Table 1:** Percentage of knee, hand, and hip osteoarthritis (OA) by each reference standard, knee OA assessment, and other baseline characteristics of 171 subjects who underwent comprehensive clinical assessment for knee OA by sex.

Characteristics	Women% (*n* = 84)	Men% (*n* = 87)	*p* value
Reference standards			
RS1	77.4	57.5	<0.01
RS2	82.1	56.3	<0.01
RS3	96.4	89.7	0.08
RS4	84.5	62.1	<0.01

Knee OA assessment			
Clinical ACR criteria	48.8	40.2	0.26
K-L grade ≥ 2	42.9	44.9	0.79
MRI cartilage score ≥ 2	91.7	88.5	0.48
Self-report	50.0	48.3	0.82

Other characteristics			
Age in years			0.44
40–49	14.3	21.8	
50–64	51.2	46.0	
65–79	34.5	32.2	
Body mass index (kg/m^2^)			0.02
18.5–24.9	46.3	28.7	
25.0–29.9	27.4	46.0	
30+	26.2	25.3	
Hip pain	42.9	18.4	<0.01
Symptomatic hand OA	43.4	18.4	<0.01

RS1, RS2, RS3, and RS4 are four reference standards including knee, hand, and hip OA which are described in Methods.

K-L = Kellgren-Lawrence, self-report = self-reported physician diagnosed knee OA, MRI = magnetic resonance imaging, and ACR = American College of Rheumatology.

**Table 2:** Validation results with the 95% confidence intervals of administrative definition of osteoarthritis using four reference standards that include knee, hand, and hip OA.

Reference standard	Administrative osteoarthritis	Sensitivity% (95% CI)	Specificity% (95% CI)	PPV% (95% CI)	NPV% (95% CI)	LR+ (95% CI)	LR− (95% CI)
RS1	AOA1	55 (45–64)	75 (61–85)	82 (71–89)	45 (34–55)	2.2 (1.4–3.6)	0.6 (0.5–0.8)
	AOA2	25 (17–34)	91 (80–97)	85 (68–94)	37 (29–46)	2.8 (1.2–6.9)	0.8 (0.7–0.9)

RS2	AOA1	55 (46–64)	77 (63–87)	84 (74–91)	44 (34–54)	2.4 (1.4–4.1)	0.6 (0.5–0.7)
	AOA2	26 (18–35)	94 (83–99)	91 (75–98)	36 (29–45)	4.6 (1.5–14.5)	0.8 (0.7–0.9)

RS3	AOA1	47 (39–55)	75 (43–93)	96 (88–99)	10 (5–18)	1.9 (0.7–5.0)	0.7 (0.5–1.0)
	AOA2	21 (15–29)	100 (70–100)	100 (87–100)	9 (5–15)	>21 (21–∞)	0.8 (0.7–0.9)

RS4	AOA1	57 (48–66)	87 (73–95)	92 (83–97)	42 (33–53)	4.4 (2.0–9.3)	0.5 (0.4–0.6)
	AOA2	26 (18–34)	96 (84–99)	94 (79–99)	32 (24–41)	6.5 (1.5–23.6)	0.8 (0.7–0.9)

AOA1 includes at least one visit to a health professional or one hospital admission for osteoarthritis and AOA2 includes at least two visits to health professionals in two years or one hospital admission for osteoarthritis. RS1, RS2, RS3, and RS4 are four reference standards that include knee, hand, and hip OA which are described in Methods.

PPV: positive predictive value; NPV: negative predictive value; LR+: positive likelihood ratio = sensitivity/(1 − specificity); LR−: negative likelihood ratio = (1 − sensitivity)/specificity.
